# Economic Burden of the 2020 COVID-19 Hospitalizations in Spain

**DOI:** 10.1001/jamanetworkopen.2022.50960

**Published:** 2023-01-13

**Authors:** Blanca Álvarez-del Río, Laura Sánchez-de Prada, Alejandro Álvaro-Meca, Marta Martín-Fernández, F. Javier Álvarez, Eduardo Tamayo, Eduardo Gutiérrez-Abejón

**Affiliations:** 1 Pharmacology Department, Faculty of Medicine, University of Valladolid, Valladolid, Spain; 2 BioCritic, Group for Biomedical Research in Critical Care Medicine, Valladolid, Spain; 3 Hospital Clínico Universitario de Valladolid, Valladolid, Spain; 4 National Influenza Centre, Valladolid, Spain; 5 Centro de Investigación Biomédica en Red de Enfermedades Infecciosas (CIBERINFEC), Instituto de Salud Carlos III, Madrid, Spain; 6 Preventive Medicine and Public Health Department, Rey Juan Carlos University, Madrid, Spain; 7 Pharmacy Directorate, Castilla y León Health Council, Valladolid, Spain

## Abstract

This economic evaluation reports the total and per patient costs of inpatient care for COVID-19 in Spain in 2020.

## Introduction

In Spain, the 2020 total health care expenditure surpassed €115 billion, covering 47.5 million people.^1^ The health care burden increased due to additional financing required to address the COVID-19 pandemic. This study aimed to describe the direct costs of COVID-19 treatment of hospitalized patients during the first year of the pandemic.

## Methods

We conducted a nationwide, population-based retrospective economic evaluation of all COVID-19–related hospitalizations in Spanish hospitals in 2020. This study was approved by the CEIm Area de Salud Valladolid Este Ethics Review Board, which waived the informed consent requirement given the anonymous character of the data used in the study. We followed the CHEERS reporting guideline.

Data were collected from the Minimum Basic Data Set (MBDS), a clinical and administrative database that covers approximately 99.5% of public and private Spanish hospitals and includes discharge reports. Patients hospitalized for COVID-19 between January 1 and December 31, 2020, were selected. The MBDS excludes short-term admissions (<24 hours), emergency department care, and outpatient care. Patients with *International Statistical Classification of Diseases Tenth Revision, Clinical Modification* codes B97.29 and U07.1 as the primary diagnosis on admission were included in the analysis (eMethods in Supplement 1).

Hospital costs were calculated using diagnosis-related group data from the MBDS and were disaggregated by sex, age group, intensive care unit (ICU) admission, outcome (death or survival), and epidemic wave (first or second). All costs were expressed in euros in 2020 values. For discussion purposes, the European Central Bank currency equivalence in 2020 was used (eMethods in Supplement 1).

Results were reported as mean (95% CI) for continuous variables and frequency (percentage) for categorical variables. Differences between groups were assessed using an unpaired, 2-tailed *t* test; Mann-Whitney test; and Kruskal-Wallis test with Bonferroni correction adjustment for multiple comparisons (α = .05) for continuous variables when appropriate. Statistical analysis was conducted with Python 3.9 (Python). Two-sided *P* < .05 indicated statistical significance.

## Results

A total of 217 106 patients with a COVID-19 diagnosis (94 542 females [43.5%], 122 564 males [56.5%]; mean [SD] age, 67.1 [22.5] years) were admitted in 2020. Admission characteristics are described in Table 1. The mean cost of admissions was €1 234 202 626.55 (total cost, €18 428 397 789.48), with a mean cost per patient of €5684.79 (Table 2) and €4880.76 for non–COVID-19 causes. The cost per wave was €623 077 956.36 in the first and €611 124 670.19 in the second, with no differences in mean cost per patient. To convert amounts to US dollars, multiply by 1.14.

**Table 1.** Characteristics of Patients With COVID-19–Related Hospitalizations in 2020

Characteristic	No. (%)^a^
No. of patients	217 106
Sex	
Female	94 542 (43.5)
Male	122 564 (56.5)
Age, mean (95% CI), y	67.1 (67.03-67.18)
Length of stay, mean (95% CI), d	10.86 (10.81-10.91)
In-hospital death	36 034 (16.6)
Charlson Comorbidity Index, mean (95% CI)	1.35 (1.35-1.36)
ICU admission and intubation	
ICU admission	19 857 (9.2)
ICU death	6294 (2.9)
ICU length of stay, mean (95% CI)	15.7 (15.47-15.93)
Mechanical ventilation	14 527 (6.7)
Ventilatory assistance	12 303 (5.7)

Abbreviation: ICU, intensive care unit.

^a^ Values are expressed as absolute number (%) for categorical variables and as mean (95% CI) for continuous variables.

**Table 2.** Costs of COVID-19–Related Hospitalization

Description	Patients, No.	Total cost, €^a^	Mean cost per patient (95% CI), €^a^
All COVID-19 hospitalizations	217 106	1 234 202 626.55	5684.79 (5648.86-5720.73)
Epidemic wave			
First	109 018	623 077 956.36	5715.37 (5658.18-5772.55)
Second	108 088	611 124 670.19	5653.95 (5610.55-5697.35)
Type of admission			
General	197 249	813 250 043.85	4122.96 (4109.15-4136.77)
ICU	19 857	420 952 582.70	21 199.20 (20 908.84-21489.56)
Outcome			
Survival	181 072	918 014 423.38	5069.89 (5035.90-5103.87)
Death	36 034	316 188 203.17	8774.72 (8646.29-8903.15)
Sex			
Male	122 564	766 173 398.20	6251.21 (6196.72-6305.70)
Female	94 542	468 029 228.35	4950.49 (4908.28-4992.70)
Age group, y			
<40	14 156	61 674 501.59	4356.77 (4259.35-4454.20)
40-59	55 971	308 345 337.62	5509.02 (5436.25-5581.79)
60-79	85 437	588 135 960.84	6883.86 (6810.12-6957.59)
>79	61 542	276 046 826.50	4485.50 (4463.81-4507.19)

Abbreviation: ICU, intensive care unit.

^a^ To convert to US dollars, multiply by 1.14.

The ICU admissions accounted for 9.5% of all admissions, with a higher mean cost per patient than general admissions (€21 199.20 [95% CI, €20 908.84-€21489.56] vs €4122.96 [95% CI, €4109.15-€4136.77]; *P* < .001). Higher mean spending per patient was found in those who died vs survived (€8774.72 [95% CI, €8646.29-€8903.15] vs €5069.89 [95% CI, €5035.90- €5103.87]; *P* < .001) (Table 2). Males had a higher mean cost per patient than females (€6251.21 €6196.72-€6305.70] vs €4950.49 [95% CI, €4908.28-€4992.70]; *P* < .001). The highest mean cost per patient was among the 60- to 79-year group (Table 2).

## Discussion

Expenditures for COVID-19-related hospitalizations in Spain amounted to €1.23 billion in 2020, which represented 6.7% of total hospital costs with a mean cost per patient that was 16% higher than for patients admitted for non–COVID-19 causes.^1^ These costs were lower than those reported in studies from Canada^2^ (US $12 728 for general and $42 340 for ICU admission), Greece ($10 110 and $27 603.54, respectively),^3^ and the US (estimated mean cost, $14 366).^4^ In contrast, costs were higher than those in Turkey ($881.75 and $2924, respectively)^3^ and Colombia^5^ (median cost, $1688). These comparisons should be interpreted with caution due to different sample sizes and methods employed.

A study limitation was the use of MBDS, which estimates patient costs through diagnosis-related group, a world-renowned and consolidated American cost-estimation system.^6^ Results of this economic evaluation may be useful to health authorities for developing an economic strategy in preparation for future pandemics.

## Notes

Supplement 1.
eMethods.

Supplement 2.
Data Sharing Statement

## References

[1] Spanish Ministry of Health. Annual report on the National Health System 2020-2021. Accessed August 8, 2022. https://www.sanidad.gob.es/estadEstudios/estadisticas/sisInfSanSNS/tablasEstadisticas/InfAnualSNS2020_21/INFORME_ANUAL_2020_21.pdf

[2] Cheung DC, Bremner KE, Tsui TCO, . “Bring the hoses to where the fire is!”: differential impacts of marginalization and socioeconomic status on COVID-19 case counts and healthcare costs. Value Health. 2022;25(8):1307-1316. doi:10.1016/j.jval.2022.03.019 35527165 PMC9072854

[3] Richards F, Kodjamanova P, Chen X, . Economic burden of COVID-19: a systematic review. Clinicoecon Outcomes Res. 2022;14:293-307. doi:10.2147/CEOR.S338225 35509962 PMC9060810

[4] Bartsch SM, Ferguson MC, McKinnell JA, . The potential health care costs and resource use associated with COVID-19 in the United States. Health Aff (Millwood). 2020;39(6):927-935. doi:10.1377/hlthaff.2020.00426 32324428 PMC11027994

[5] Alvis-Zakzuk NJ, Flórez-Tanus Á, Díaz-Jiménez D, . How expensive are hospitalizations by COVID-19? Evidence from Colombia. Value Health Reg Issues. 2022;31:127-133. doi:10.1016/j.vhri.2022.04.005 35671540 PMC9167033

[6] Gluckman TJ, Spinelli KJ, Wang M, . Trends in diagnosis related groups for inpatient admissions and associated changes in payment from 2012 to 2016. JAMA Netw Open. 2020;3(12):e2028470. doi:10.1001/jamanetworkopen.2020.28470 33284340 PMC12527476

