# Supplementary material for: Economic Burden of the 2020 COVID-19 Hospitalizations in Spain
Source: JAMA Netw Open. 2023 Jan 13;6(1):e2250960. doi: 10.1001/jamanetworkopen.2022.50960 (PMC9857216; doi:10.1001/jamanetworkopen.2022.50960)
Supplement: Supplement 1. — eMethods. [file jamanetwopen-e2250960-s001.pdf]

Supplementary Online Content

Álvarez-del Río B, Sánchez-de Prada L, Álvaro-Meca A, et al. Economic burden of the 2020 COVID-19 hospitalizations in Spain. *JAMA Netw Open*. 2023;6(1):e2250960. doi:10.1001/jamanetworkopen.2022.50960

eMethods.

This supplementary material has been provided by the authors to give readers additional information about their work.

© 2023 Álvarez-del Río B et al. *JAMA Network Open.*

eMethods

Study design

A nationwide population-based retrospective study of all hospitalizations involving COVID-19 was carried out in Spanish hospitals (public and private) during the first year of the SARS-CoV2 pandemic, 2020.

Data were collected from the Minimum Basic Data Set (MBDS)[1] obtained by the National Surveillance System for Hospital Data in Spain and published by the Ministry of Health. The MBDS is a clinical and administrative data base fulfilled at the time of discharge with an estimated coverage of 99.5% of public and private Spanish hospitals. It provides encrypted information following the International Classification of Diseases 10th Revision, Clinical Modification (ICD-10-CM)[2] The data were treated with full confidentiality according to Spanish legislation. The study was approved by the Ethics Review Board (CEIm Area de Salud Valladolid Este, reference study PI 22-2855).

Measures

Hospitalized patients during 2020 were selected. The MBDS excludes programmed short-term admissions (less than 24 hours), emergency room and hospital outpatient care. COVID-19 patients with ICD-10-CM[3] codes B97.29 and U07.1 as main diagnosis present on admission were selected.

Variables collected included age, sex, length of stay (LoS), in-hospital death, admission in intensive care unit (ICU), ICU length of stay, ICU death, mechanical ventilation and ventilatory assistance. Charlson comorbities index was computed using ICD-10 coding algorithms developed by Quan et al.[3] During 2020, SARS-CoV2 circulated in two waves in Spain, the first one since its emergence until June 30th, 2020, and the second from July 1st, 2020, until December 31st, 2020.[4]

COVID-19 related hospital costs were calculated using diagnosis-related groups (DRG) data extracted from the MBDS.[1] These costs were disaggregated based on sex, age groups (<40, 40-59, 60-79, >79 years old), ICU-admission, outcome (death/alive) and epidemiological waves (first and second). All costs were expressed in euros (€) in 2020 values. For discussion purposes the annual currency equivalence in 2020 was 1€ =1.1422$ United States dollars (USD)[5]

Statistical analysis

Results were reported as mean (95% confidence interval) for continuous variables and as frequencies and percentages for categorical variables. Differences between groups were assessed using an unpaired, 2-tailedt test; Mann-Whitney test; and Kruskal-Wallis test with Bonferroni correction adjustment for multiple comparisons ($\alpha = 0.05$) for continuous variables when appropriate. Statistical analysis was conducted with Python 3.9 (Python). (Two-sided $P < .05$ indicated statistical significance).

eReferences

1. Spanish Ministry of Health. Specialized Care Register (SCR-MBDS). Activity and results of hospitalizations in Spain. Published online 2022. Accessed August 8, 2022. https://pestadistico.inteligenciadegestion.sanidad.gob.es/publicoSNS/N/rae-cmbd/rae-cmbd

2. Spanish Ministry of Health. ICD-10 regulations. MBDS. COVID-19 coding regulations. Published online 2020. Accessed August 8, 2022. https://www.sanidad.gob.es/estadEstudios/estadisticas/normalizacion/CIE10/PREGUNTAS_UT_Covid_19_ms_070420202.pdf

3. Quan H, Sundararajan V, Halfon P, et al. Coding algorithms for defining comorbidities in ICD-9-CM and ICD-10 administrative data. Med Care. 2005;43(11):1130-1139. doi:10.1097/01.mlr.0000182534.19832.83

4. Spanish Ministry of Health. Annual Report on the National Health System 2020-2021. Published online 2022. Accessed August 8, 2022. https://www.sanidad.gob.es/estadEstudios/estadisticas/sisInfSanSNS/tablasEstadisticas/InfAnualSNS2020_21/INFORME_ANUAL_2020_21.pdf

© 2023 Álvarez-del Río B et al. *JAMA Network Open.*

5. European Central Bank. Eurosystem policies and exchange rates. Accessed November 14, 2022. https://www.ecb.europa.eu/stats/policy_and_exchange_rates/euro_reference_exchange_rates/html/eurofxref-graph-cad.en.html

© 2023 Álvarez-del Río B et al. *JAMA Network Open.*
