# Supplementary material for: Economic Burden of the 2020 COVID-19 Hospitalizations in Spain
Source: JAMA Netw Open. 2023 Jan 13;6(1):e2250960. doi: 10.1001/jamanetworkopen.2022.50960 (PMC9857216; doi:10.1001/jamanetworkopen.2022.50960)
Supplement: Supplement 2. — Data Sharing Statement [file jamanetwopen-e2250960-s002.pdf]

Data Sharing Statement

Álvarez-del Río. Economic Burden of the 2020 COVID-19 Hospitalizations in Spain. *JAMA Netw Open*. Published January 13, 2023. doi:10.1001/jamanetworkopen.2022.50960

Data
Data available: No

Additional Information
Explanation for why data not available: The MDBS is the property of the Ministry of Health. Therefore, any researcher can request the data related to this article from the Ministry of Health by email (icmbd@msssi.es), by fax (+34915964111), or by mail (Instituto de Información Sanitaria, Área de Información y Estadísticas Asistenciales, Ministerio de Sanidad, Consumo y Bienestar Social. Paseo del Prado 18-20; 28071 Madrid. Spain).
